# Attractor-state itinerancy in neural circuits with synaptic depression

**DOI:** 10.1186/s13408-020-00093-w

**Published:** 2020-09-11

**Authors:** Bolun Chen, Paul Miller

**Affiliations:** 1 Volen National Center for Complex Systems, Brandeis University, Waltham, MA 02453 USA; 2 Department of Biology, Brandeis University, Waltham, MA 02453 USA

**Keywords:** Attractors, Bistability, Synaptic depression

## Abstract

Neural populations with strong excitatory recurrent connections can support bistable states in their mean firing rates. Multiple fixed points in a network of such bistable units can be used to model memory retrieval and pattern separation. The stability of fixed points may change on a slower timescale than that of the dynamics due to short-term synaptic depression, leading to transitions between quasi-stable point attractor states in a sequence that depends on the history of stimuli. To better understand these behaviors, we study a minimal model, which characterizes multiple fixed points and transitions between them in response to stimuli with diverse time- and amplitude-dependencies. The interplay between the fast dynamics of firing rate and synaptic responses and the slower timescale of synaptic depression makes the neural activity sensitive to the amplitude and duration of square-pulse stimuli in a nontrivial, history-dependent manner. Weak cross-couplings further deform the basins of attraction for different fixed points into intricate shapes. We find that while short-term synaptic depression can reduce the total number of stable fixed points in a network, it tends to strongly increase the number of fixed points visited upon repetitions of fixed stimuli. Our analysis provides a natural explanation for the system’s rich responses to stimuli of different durations and amplitudes while demonstrating the encoding capability of bistable neural populations for dynamical features of incoming stimuli.

## Introduction

Mounting evidence suggests that neural ensembles can give rise to states of activity that are stable and attractor-like over a short period [1–8]. However, given the range of timescales of neural processes, either slower processes or intrinsic noise typically ensures that an activity state does not remain stable for more than a few hundred milliseconds, even when a stimulus is constant. For example, when viewing images that can give rise to bistable percepts, a switching between the distinct perceived images arises [2, 4, 6, 7]. A similar switching can arise with auditory stimuli [1]. Analysis via hidden Markov modeling [9–15] or change-point methods [16, 17] has suggested such state-switching in neural activity in sensory and decision-related tasks. Modeling work has shown how discrete attractor states can arise, and how either noise [18–22], or slow adaptation-like processes such as synaptic depression [23, 24], or a combination of the two [25, 26], can lead to transitions between these states, which we refer to as quasi-stable attractor states [8].

In this article we focus on how short-term synaptic depression [27–29] can lead to the instability of one quasi-stable attractor state, inducing a transition to a new state, which itself may be stable or quasi-stable. Neuronal populations with short-term synaptic depression have been studied extensively. Spontaneous activity in the auditory cortex can be model by a spatial firing rate model as a result of dynamical synapse [30]. Holcman and Tsodyks [31] considered a single rate model with slow synaptic depression. With varying synaptic coupling weights, the UP and DOWN states are interpreted as fixed points. State transitions can be triggered by noisy fluctuations. The population exhibits a state-dependent response to a constant stimulus. Barak and Tsodyks [32] performed a fast-slow analysis on a rate model with both short-term facilitation and depression. They obtained a bifurcation diagram for the synaptic strength vs facilitation index. They found that facilitation enables a slow and reversible transition to persistent firing. Depression, on the other hand, leads to a rapid and transient increase in activity, which was referred to as “population spikes”. Melamed et al. [33] examined slow oscillations (below 5 Hz) induced in an E-I rate model with facilitating E to I couplings. They focused on oscillations between UP and DOWN states. It was shown that the oscillation frequency depends on the synaptic time constant and the coupling strength in a pair of E-I populations. Moreover, a thorough bifurcation analysis done in Ref. [34] links the stability of UP state to the rich pattern-formation in a two-dimensional neural field with synaptic depression. Here we focus on the history dependence of the response of circuits with many such states (arising from multiple bistable units) in response to a simple input. However, to provide some insight into the mechanism, we begin with a description of the behavior of a single unit and two coupled units in places reiterating the results of others.

Mathematically, if one fixes the amount of synaptic depression by setting a slow, synaptic depression variable to a constant, groups of neurons with strong self-feedback can possess multiple stable discrete attractor states. The system can resemble a relaxation oscillator with sufficiently strong depression and feedback [35] as the depression variable slowly decreases for an active group of neurons, reducing the within-group effective excitatory coupling until the activity can no longer be maintained. Once inactive, the depression variable slowly recovers, allowing for connections to re-strengthen and activity to recommence. In other ranges of parameters, the remnant of such potential oscillatory behavior leads to a rich repertoire of states and state transitions in response to simple stimuli when the stable states of such systems are fixed points.

We characterize such systems with small numbers of potentially bistable groups of neurons via the number of stable fixed points and their basins of attraction. The stable steady states can be used to encode information arriving at the circuit via stimuli with varying duration and amplitude [36]. So the number of discrete attractor states and the state-transition sequence in response to stimuli provide measures of a network’s ability to store dynamical features of stimuli. Therefore, we assess how different fixed points are reached as a function of the amplitude or duration of stimuli, as well as the system’s state before stimulus onset. In particular, we use an extended Wilson–Cowan model [37] and incorporate synaptic depression to show how weak coupling between distinct bistable populations impacts the states’ basins of attraction, which can be deformed into complex shapes. In so doing, we offer an initial explanation of the rich information processing capabilities of high-dimensional networks with multiple attractor states and slow synaptic dynamics.

The rest of this paper is organized as follows: We introduce the rate model with synaptic depression in Sect. 2 and derive the dimensionless form that will be used for later analysis. In Sect. 3, we numerically explore the dynamics of small networks whose responses to constant inputs exhibit history dependence. As system size increases, the synaptic depression enables the network to traverse more states and form longer transition sequences under repetitive stimulations. We summarize in Sect. 4 and discuss some open questions for future research.

## Model

We consider a network of *N* neural populations, each of which can be characterized by its mean firing rate $r_{i}$. The dynamics of the population rate $r_{i}$ in response to time-varying current $I_{i} (t )$ is given by a generic form:

1 $$ \begin{aligned} &\tau _{r}\dot{r}_{i} = -r_{i}+ \frac{r_{i}^{\max }}{1+\exp [- (I_{i} (t )- \Theta _{i} )/\Delta _{i} ]}, \\ &I_{i} (t ) = \sum_{j=1}^{N}W_{ij}s_{j}+I_{i}^{{\mathrm{app}}} (t ). \end{aligned} $$

Here $r_{i}^{\max }$ is the maximum firing rate, $\Theta _{i}$ is the input threshold for the half-maximum firing rate, and $\Delta _{i}$ is inversely proportional to the slope of the input-output curve. The input current $I_{i} (t )$ consists of two parts: (1) synaptic currents from the network with a connectivity $W_{ij}$ which quantifies the coupling strength from population *j* to population *i*; (2) an applied current $I_{i}^{{\mathrm{app}}} (t )$.

The time-varying effective synaptic input $s_{i}$, arising from a population *i*, is given as a fraction of the maximum possible (so $s_{i}\in [0,1 ]$). We assume spikes are emitted from the population via a Poisson process and include a short-term synaptic depression factor $d_{i}\in [0,1 ]$, with 0 indicating a fully depressed synapse. With these assumptions, the mean dynamics of $s_{i}$ and $d_{i}$ take the following form [24]:

2 $$\begin{aligned}& \tau _{s}\dot{s}_{i} = -s_{i}+\rho p_{0}r_{i}d_{i}\tau _{s} (1-s_{i} ), \end{aligned}$$

3 $$\begin{aligned}& \tau _{d}\dot{d}_{i} = 1-d_{i}-p_{0}r_{i}d_{i} \tau _{d}. \end{aligned}$$

The parameter $p_{0}$ gives the fraction of docked vesicles released per spike. *ρ* is the fraction of open receptors bound by maximal vesicle release such that $\rho p_{0}d_{i}$ is the fraction of closed synaptic receptors that open, so it is proportional to the increase in the synaptic current for a given presynaptic spike.

The time constants for the mean firing rate, the synaptic current, and the depression variable are denoted respectively as $\tau _{r}$, $\tau _{s}$, and $\tau _{d}$. Since these dynamical variables vary over distinct time scales, it is convenient to rescale the time and to normalize the rate: $t/\tau _{r}\to t$, $r_{i}/r_{i}^{\max }\to r_{i}\in [0,1 ]$, as well as to scale the input and threshold by $\Delta _{i}$: $I_{i}^{\mathrm{app}}/\Delta _{i}\to I_{i}$, $W_{ij}/\Delta _{i}\to w_{ij}$, and $\Theta _{i}/\Delta _{i}\to \theta _{i}$. The dimensionless equations then become

4 $$\begin{aligned}& \dot{r}_{i} = -r_{i}+f \Biggl(\sum _{j=1}^{N}w_{ij}s_{j}- \theta _{i}+I_{i} (t ) \Biggr), \end{aligned}$$

5 $$\begin{aligned}& \dot{s}_{i} = \alpha \bigl(-s_{i}+br_{i}d_{i} (1-s_{i} ) \bigr), \end{aligned}$$

6 $$\begin{aligned}& \dot{d}_{i} = \beta (1-d_{i}-ar_{i}d_{i} ), \end{aligned}$$

where $f (x )= (1+e^{-x} )^{-1}$ is the logistic function, $\theta _{i}$ is the activation threshold. The weight matrix $w_{ij}$ determines the coupling strengths within a unit and between units. Two remaining time-scales are characterized by $\alpha =\tau _{r}/\tau _{s}$ and $\beta =\tau _{r}/\tau _{d}$. In this paper, we assume that the short-term depression varies over a slow time scale compared with the firing rate and the synaptic current. This situation arises when the timescale for recovery from depression is significantly longer than other time constants, such that $\tau _{d}\gg \tau _{s}>\tau _{r}$. For example, we set $\tau _{d}=250\text{ ms}$, $\tau _{s}=50\text{ ms}$, and $\tau _{r}=10\text{ ms}$ in simulations, and $p_{0}=0.5$. The dimensionless parameters

7 $$ a =p_{0}r_{i}^{\max }\tau _{d}, \qquad b =\rho p_{0}r_{i}^{\max } \tau _{s} $$

quantify the degree of synaptic depression and the amplitude of synaptic currents, respectively. With slow depression, $a > b$.

Finally, all cell groups are assumed to be comprised of neurons with identical parameters. For most simulations we choose the standard parameter set: $a=6.25$, $b=1.25$, $w_{ii}=40$, and $\theta _{i}=5$, unless noted otherwise. In a control scenario [for example, Fig. 2(b1)–(b4)], to demonstrate the importance of synaptic depression, we produce a network without depression by setting $\tau _{d}\to 0$ thus $a\to 0$ and $d_{i}\to 1$. Then the firing rate is solely driven by the synaptic current within a time window of $\tau _{s}$.

## Dynamics

The fixed point solution of *N* coupled units satisfies

8 $$ g (r_{i} )-\sum_{j=1}^{N}w_{ij}s (r_{j} )=I_{i}- \theta _{i}, $$

where $g (r )=f^{-1}(r)=\ln [r/ (1-r ) ]$ and $s (r )=\frac{br}{1+ (a+b )r}$ is the steady synaptic current. At a fixed point $r= (r_{1},\ldots ,r_{N} )^{T}$, the steady values of *s* and *d* are given by Eqs. (5) and (6). Linearization at the fixed point leads to a blocked Jacobian matrix

9 $$ J_{ij}= \begin{bmatrix} -\delta _{ij} & w_{ij}r_{i} (1-r_{i} ) & 0 \\ \delta _{ij}\frac{\alpha b}{1+(a+b)r_{i}} & -\delta _{ij} \frac{\alpha (1+(a+b)r_{i})}{1+ar_{i}} & \delta _{ij} \frac{\alpha br_{i}(1+ar_{i})}{1+(a+b)r_{i}} \\ -\delta _{ij}\frac{\beta a}{1+ar_{i}} & 0 & -\delta _{ij}\beta (1+ar_{i}) \end{bmatrix}. $$

Here, $\delta _{ij}$ is the Kronecker delta, $i,j=1,\ldots ,N$.

A hyperbolic fixed point is a saddle with degree *k* ($k=0,1,\ldots ,N$) if there are *k* eigenvalues of the Jacobian with positive real parts ($\operatorname{Re}\lambda _{i}>0$, ∀*i*). Strong self-excitation can make a single unit bistable (coexistence of two stable nodes and a saddle). For *N* non-interacting bistable units, the number of saddles with degree *k* is $n_{k}=\binom{N}{k}2^{N-k}$, which is choosing *k* positive real eigenvalues out of *N* eigenvalues and multiplying the number of remaining $(N-k)$ bistable states. The total number of fixed points is then $\sum_{k=0}^{N}n_{k}=3^{N}$. Since our focus is on the number of stable states reached in response to successive stimuli, we are primarily concerned with the stability of each fixed point. While the imaginary parts of the eigenvalues of the Jacobian indicate whether a fixed point is approached as a spiral (in an oscillatory manner), such transient behavior does not impact its stability. Therefore, when counting the number of steady states, it suffices to consider only the real parts of the eigenvalues.

For *N* weakly-coupled bistable populations, the number of saddles grows quickly as *N* increases, and it outnumbers the stable nodes. The large number of saddles can give rise to heteroclinic sequences or orbits, and therefore more oscillatory firing-rate behavior. When *N* is large, the competition between intra- and inter-population couplings leads to chaotic behaviors [38]. The rich structure of attractors defines a dynamical “landscape” of the neural activity. It is worth mentioning that we consider small networks with weak recurrent connections, which correspond to the multi-stable region in Ref. [38]. Strong cross-connections inevitably cause stable fixed points to destabilize or to disappear via merging with unstable fixed points. Therefore, there is a tradeoff between the richness gained with random cross-connections and the reduction in the number of stable states that can result.

In this section, we examine the network’s response to constant and repetitive stimuli. We show that short-term synaptic depression and weak inter-population couplings facilitate transitions among multiple fixed points.

### History-dependent responses to stimuli

The rich dynamical response of a single population has been first observed and systematically discussed in earlier works [31, 32]. Here we revisit the problem focusing on the history dependence under a stimulus

10 $$ I (t )=I_{\mathrm{app}} \bigl[H (t-t_{0} )-H (t-t_{0}- \tau _{\mathrm{dur}} ) \bigr], $$

where *H* is the Heaviside step function, $I_{\mathrm{app}}$ is the amplitude, $\tau _{\mathrm{dur}}$ is the duration, and $t_{0}$ is the onset time.

**Figure 1:**
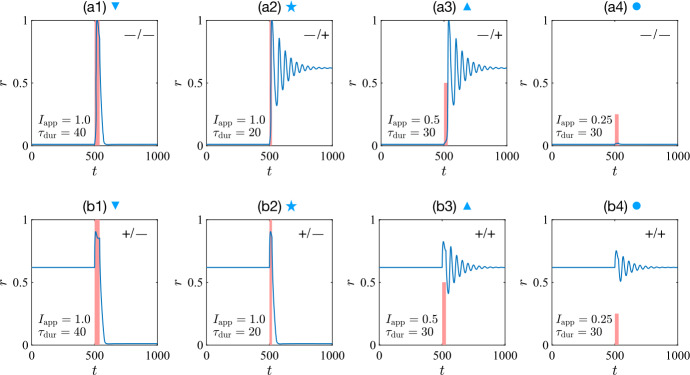
The firing rate of a bistable unit evolves under constant stimuli (red bars) with different durations and amplitudes. The final states depend on initial states, amplitudes, and durations, with symbol “+” standing for the ON state and “–” for the OFF state. Panels (**a1**)–(**b1**), marked by a down-triangle, show no history dependence. Panels (**a2**)–(**b2**), marked by a star, show maximal history dependence. Panels (**a3**)–(**b3**), marked by an up-triangle, again show no history dependence as the final state is independent of the initial state. Panels (**a4**)–(**b4**), marked by a circle, show the trivial history dependence of a bistable system, as with the amplitude halved from that used in (**a3**) and (**b3**) the stimulus causes no change in state. These markers correspond to different regions in the phase diagram [see Fig. 2(a3)–(a4)]

In the presence of synaptic depression, the final state not only depends on the stimulus duration and amplitude, but also on the initial state; for instance, in Fig. 1, a constant stimulus is given at $t_{0}=500$. The initial state of the bistable unit can be either OFF (marked by “–”) or ON (marked by “+”). The unit approaches different final states after the stimulus, exhibiting four types of responses: OFF-to-OFF (“–/–”), OFF-to-ON (“–/+”), ON-to-OFF (“+/–”), and ON-to-ON (“+/+”).

Upon receiving a second stimulus, it should be noted that only one combination, OFF-to-ON (a2) and ON-to-OFF (b2), which are marked by stars in Fig. 1, is maximally history-dependent in the manner we are interested in, since the same stimulus can induce two different types of switch. Such state-dependent and hence history-dependent switches will lead to itinerancy in a larger system. Meanwhile, OFF-to-ON (a3) and ON-to-ON (b3) transitions indicate the system is bistable, a fact that also leads to a trivial history dependence in that a small stimulus does not cause a state transition (a4)–(b4).

**Figure 2:**
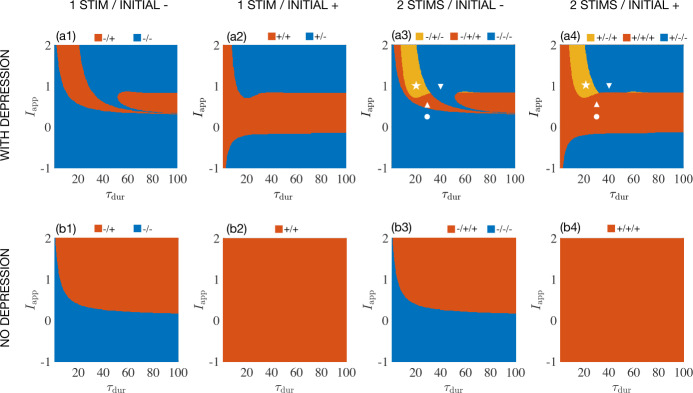
Final states of a single unit show history dependence for certain durations $\tau _{\text{dur}}$ and amplitudes $I_{\text{app}}$ of an applied stimulus. Rows compare the effects with (row a) and without (row b) short-term synaptic depression. Columns differ by whether the initial state of the system is the ON state (columns 1 and 3) or the OFF state (columns 2 and 4). Columns 1 and 2 depict the results of a single stimulus presentation, while columns 3 and 4 depict the results of a sequence of two stimulus presentations. In columns 1 and 2, the final state is color-coded as red=ON and blue=OFF. In columns 3 and 4 the red region and blue region indicate the final state is ON (red) or OFF (blue) while the yellow regions marked by a white star show maximal history dependence where the input repeatedly switches the state back and forth. Markers correspond to simulations in Fig. 1. No such repeated switching is observed without synaptic depression in panels (**b3**) and (**b4**)

Figure 2(a1) shows the final state as a function of the duration $\tau _{{\mathrm{dur}}}$ and amplitude $I_{{\mathrm{app}}}$ of the applied stimulus. The top row indicates a unit with synaptic depression, while the bottom row indicates a unit without synaptic depression. The final state reached from a single pulse when the system starts in the OFF state (column 1) can be different from the final state when we start with an ON state (column 2). [Fig. 2(a2)]. For some values of $\tau _{{\mathrm{dur}}}$ and $I_{{\mathrm{app}}}$ [yellow regions in Fig. 2(a3) and (a4)], the state of unit switches twice when applying two identical stimulations. Note that there is also a second yellow region around $(\tau _{{\mathrm{dur}}}\approx 60, I_{{\mathrm{app}}}\approx 1 )$. As mentioned in the above, this switching behavior implies that the system’s responses to constant stimuli are history-dependent. The key ingredient here is the synaptic depression. If there is no depression, as shown in Fig. 2(b3) and (b4), there is either only a single transition possible between the ON and the OFF states, or only a single stable state. The unit never switches back and forth under repetitive stimulations.

### Basins of attraction deformed by cross-couplings

Even weak inter-population couplings may deform the attracting basins of fixed points by creating new attractors and annihilating old ones. Shapes and sizes of basins defines the landscape in the state space, which affects how the system traverses through attractor-states before settling to a final state. When a stimulus is applied, the whole landscape shifts. The system’s state at the onset time (the history), the stimulus, and the geometry of basins (due to depression and couplings) jointly determine the evolution. This geometric perspective provides a natural explanation of the history-dependent responses.

Take a two-unit system as an example, when the cross-coupling is zero ($w_{ij}=0$), there are four stable fixed points: both units are OFF, $(0,0 )$; both are ON, $(1,1 )$; one is OFF and the other is ON, $(0,1 )$ and $(1,0 )$. Any initial condition converges to one of the four states as its final stable state. Weak coupling ($w_{ij}\ll w_{ii}$) may both change the number of fixed points and their stability. The number and sizes of basins also change.

**Figure 3:**
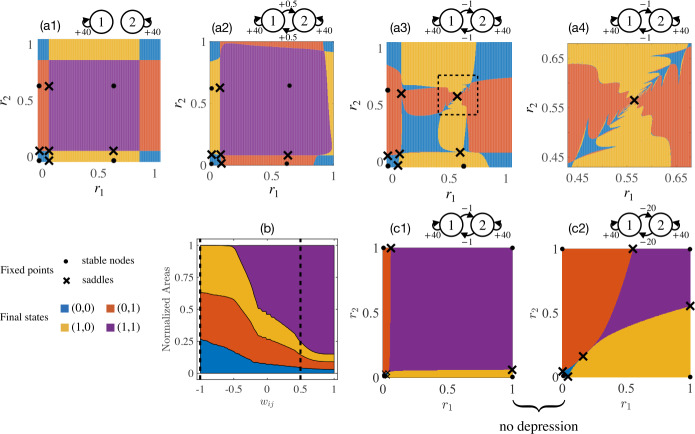
Fixed points and projected basins of attraction for two symmetrically coupled units ($w_{12}=w_{21}$). (**a1**)–(**a4**): Axes indicate the initial state of the system, with colors indicating the final state to which the system converges. Stable nodes (saddles) are labeled as filled circles (crosses) and surrounded by color-coded basins. The self-coupling is fixed at $w_{11}=w_{22}=40$ and cross-couplings are chosen as (**a1**) $w_{12}=0$, (**a2**) $w_{12}=0.5$, (**a3**)–(**a4**) $w_{12}=-1$. (**a4**) Zoom-in details reveal fine structures near the $(1,1 )$ state that is enclosed by a dashed square in (**a3**). (**b**) Normalized basin areas of stable attractors as a function of symmetric cross-coupling strength. Dashed vertical lines correspond to cases (**a2**) and (**a3**). (**c1**)–(**c2**): Labels are same as (**a1**)–(**a4**) except for the absence of depression

Figure 3 shows fixed points of two symmetrically coupled units ($w_{12}=w_{21}$) with zero input and projected basins in the $r_{1}$-$r_{2}$ plane.^1^ While cross excitations are enlarging the basin of the $(1,1)$ state (purple region), cross inhibitions quickly shrink it. When $w_{12}=w_{21}=-0.5$, the $(1,1)$ state turns into a saddle, around which the remaining basins deform into a complex structure. The final state thus would depend sensitively on the initial state, as well as and the duration and amplitude of a stimulus. Clearly, weak coupling can destabilize fixed points and thus reduce the number of stable nodes. For example, in Fig. 3(a3), under weak mutual inhibition, the $(1,1)$ state turns into a saddle with an intricate basin. Also from Fig. 3(b), the areas of basins change as a function of the cross-coupling strength. It can be anticipated that with greater excitatory cross-connection strength the $(0,0)$ state will shrink and disappear in a saddle-node bifurcation. For large $w_{ij}$, the $(1,1)$ state will be the only stable state left.

Subplots in Fig. 3(c1)–(c2) illustrate that without depression, stable fixed points have regular-shaped basins of attraction. Note that (c1) has the same coupling weights as in (a3) and (a4), except for the depression variable $d=1$. In (c2), even strong mutual inhibition ($w_{ij}=-20$) does not distort the attracting basins.

### More reachable states due to depression

We have seen that weak cross-couplings may reduce the number of stable fixed points from the $2^{N}$ available in the non-interacting system, suggesting they may decrease the information capacity of a network. However, our results suggest that the cross-couplings could lead to nontrivial dynamics, allowing for an increase in the network’s capacity to represent temporal features of stimuli. Here we explore the responses of a network to a sequence of constant stimuli, by measuring the number of final stable states reached after uniform perturbations applied to all units. This number reflects the network’s capacity to encode and maintain information about the number of stimuli it has received.

**Figure 4:**
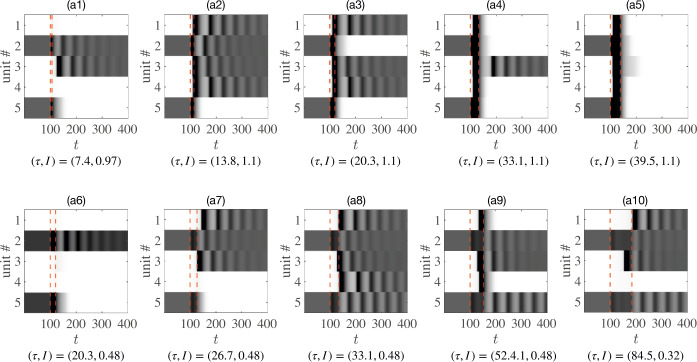
An example set of simulations of a single network of five weakly-coupled units receiving the same uniform stimulus. The duration *τ* and amplitude *I* of the stimulus differs across simulations as indicated, leading to distinct final stable states. Random coupling weights are drawn from a Gaussian distribution with a zero mean and a standard deviation of 0.1. (**a1**)–(**a10**): Evolution of five units under different stimuli starting with initial state $(01001 )$. Black (white) bars represent high (low) firing rates. Red dashed lines indicate constant stimuli received by all units

Figure 4 shows how the final stable state reached by a circuit of five weakly-coupled units can vary according to the amplitude and duration of uniform input provided to all units. Within the circuit, the cross coupling weights $w_{ij}$ are drawn from a Gaussian distribution with $\langle w_{ij} \rangle =0$ and ${\mathrm{std}} (w_{ij} )=0.1$. Other parameters are chosen such that each unit is bistable when isolated. Ranging from sharp pulses to sustained currents [Fig. 4(a1)–(a10)], different combinations of durations and amplitudes drive the same initial state^2^$(01001 )$ into ten final states: $(01101 )$, $(11110 )$, $(10110 )$, $(00100 )$, $(00000 )$, $(01000 )$, $(11100 )$, $(11111 )$, $(01101 )$, and $(11101 )$.

We next wished to assess how the number of final states reachable by application of a uniform stimulus (a box-car stimulus applied equally to all units) depended on the parameters used to produce small networks. To this end, we produced multiple instantiations of networks using random weight matrices. For each network ($w_{ij}$ fixed), the total number of stable fixed points can be calculated. We perturb an initial state $(01001 )$ by applying constant inputs equally to all units of the network and count the number of distinct steady states after the simulation (i.e., the number of reachable final states) when we vary duration and amplitude of the stimulus. We average across networks to obtain expectation values of the total fixed point number and the number of reachable states as functions of the mean *μ* and the standard deviation *σ* of the cross-connections $w_{ij}$. Moreover, we go on to assess how these results depend on the inclusion of short-term synaptic depression in our simulations. Specifically, we consider three cases: (1) strong self-coupling ($w_{ii}=40$) with depression, (2) strong self-coupling ($w_{ii}=40$) without depression, and (3) medium self-coupling ($w_{ii}=20$) without depression.

**Figure 5:**
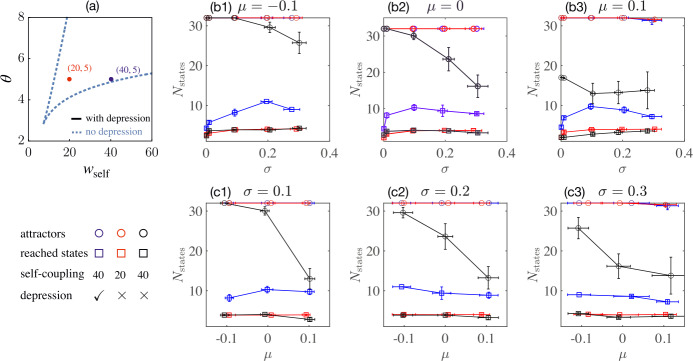
Total number of attractors (open circles) of a five-population network and the number of final states (open squares) reachable after perturbing an initial state $(01001 )$ with single square-pulse stimuli, applied equally to all units. Panel (**a**) shows bistable regions in the $w_{{\text{self}}}$-*θ* plane with and without depression, enclosed by solid black lines and dashed blue lines, respectively. The blue and red dots indicate parameters $(w_{{\text{self}}},\theta )$ used to perform the simulations. The table below panel (**a**) lists the color codes for the three cases: (1) $w_{{\text{self}}}=40$ with depression (blue), (2) $w_{{\text{self}}}=20$ without depression (red), and (3) $w_{{\mathrm{self}}}=40$ without depression (black). Panels (**b**) and (**c**) show trial-averages of total attractor numbers and the number of reachable states as functions of the mean $\mu = \langle w_{ij} \rangle $ and the standard deviation $\sigma ={\text{std}} (w_{ij} )$

As shown in Fig. 5, the circuits with strong self-coupling plus depression (case 1) outperform the other two cases in the number of reachable final states (open squares) across a broad range of parametric variation of the random cross coupling matrix. Networks without synaptic depression and medium self-coupling (case 3, Fig. 5, red open circles) have the same number of total fixed points in circuits with the relatively weak cross-connections tested here. Indeed, since depression can destabilize active states, the total number of stable fixed points can be greater in networks without depression in many other parameter ranges (data not shown). However, the networks without depression have a far smaller repertoire of final states reachable by presentation of uniform stimuli. The networks with strong self-coupling without depression (case 2) have the poorest performance in both measures, primarily because the networks are near the edge of their bistable region.

Intuitively, by reducing the effective synaptic strength of recurrent synapses of active units, synaptic depression makes it much easier for a stimulus which has activated a unit to subsequently inactivate the same unit (Figs. 1 and 2). Such non-monotonic responsiveness to stimuli at the single-unit level also revealed that the intricate structure of the basins of attraction of two coupled units (Fig. 3) enhances the repertoire of states reached by repeated stimuli when synaptic depression is included. Adaptation currents would have a very similar impact.

These results indicate that strong self-coupling combined with synaptic depression provides an underlying mechanism for attractor itinerancy, because extension of the duration of a stimulus more often causes transitions of the network’s activity to a new basin of attraction, leading to a new final stable state. That is, the duration-dependence of the final state, most evident in networks with synaptic depression, is an indication of attractor-state itinerancy.

### Repeated stimuli cause transitions through sequences of distinct states

**Figure 6:**
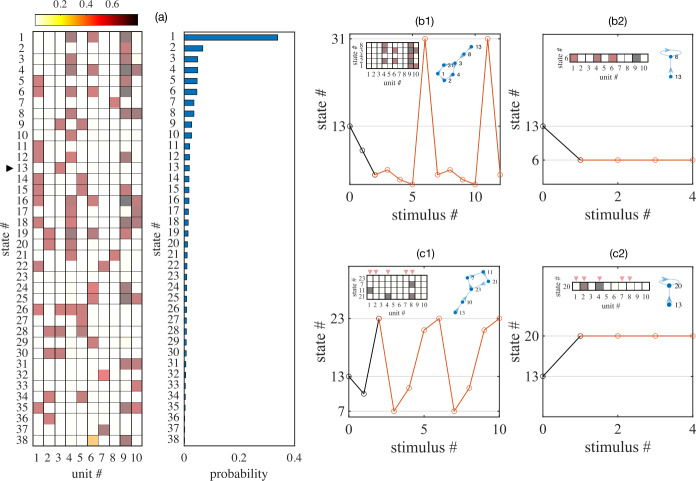
An example simulation of a randomly connected network of ten units ($\langle w_{ij} \rangle =-0.2$, ${\text{std}}(w_{ij})=1$) responding to a series of repeated identical stimuli. Panel (**a**): Firing rate profiles (left) of 38 stable fixed points sorted according to occurrence frequency (probability bar chart, right). Panels (**b1**) and (**b2**): Sequences of states reached following repeated identical stimuli ($\tau _{{\text{dur}}}=20$, $I_{{\text{app}}}=0.8$) with depression (**b1**) and without depression (**b2**) when the initial state is #13 (marked by a black triangle in (**a1**)). Red open circles indicate cycles (**b1**) or steady state (**b2**). Insets include a visualization of the steady firing rate pattern and a directional graph depicting the transition sequence. Panels (**c1**) and (**c2**): Same as panels (**b1**) and (**b2**) with only 5 randomly chosen units (marked by red triangles), instead of all of them, receiving the series of identical stimuli

In this section, to highlight the history dependence of the attractor-state itinerancy observed in these networks, we examine the networks response to a series of repeated stimuli. As an illustration, let us consider a randomly connected network of ten units receiving such a train of identical inputs. The network’s stable fixed points, as well as its basins of attraction, provide key information for estimating the sequences of states. Thanks to the relatively small size of the system, it is feasible to find all of its stable fixed points. The frequency of occurrence of a given fixed point can be viewed as the probability of finding it in the state space, which is inversely proportional to the size of its attracting basin. Figure 6(a) lists all 38 stable fixed points in a particular ten-unit network with $\langle w_{ij} \rangle =-0.2$ and ${\mathrm{std}} (w_{ij} )=1$, sorted according to their frequency of occurrence.

To explore this non-autonomous system, we start with each one of the fixed points and apply a train of constant stimuli with fixed duration ($\tau _{{\mathrm{dur}}}=20$) and amplitude ($I_{{\mathrm{app}}}=1$). Subsequent stimuli are separated by $\tau =1000$ time steps to make sure transients are completely settled. We then follow every trajectory in the state space and perform statistics on the number of unique states along trajectories.

Figure 6(b) and (c) illustrates that such trajectories originated from state 13 (marked by a black triangle in (a1)) in two scenarios:

In (b1) and (b2), all ten units receive the same stimuli in circuits with depression (b1) and without depression (b2). In response to the periodic perturbation, the network with depression (b1) falls into a stable cycle, $13\to 8\to (3\to 4\to 2\to 1\to 31 )$ with a length of five, whereas the network without depression (b2) quickly converges to a steady state (state 6).

In (c1) and (c2), only five randomly chosen units (1, 2, 4, 7, 8) out of the ten units receive the inputs. The randomness induces a period-4 sequence in the network with depression (c1): $13\to 10\to (23\to 7\to 11\to 21 )$. But in (c2) when the depression is absent, the network settles down to a steady state (state 20) after one stimulus. Notice that in both cases, some targeted units get suppressed by weak cross inhibitions. For other random subsets (data not shown here), non-targeted units can be excited due to reciprocal connections in the network.

**Figure 7:**
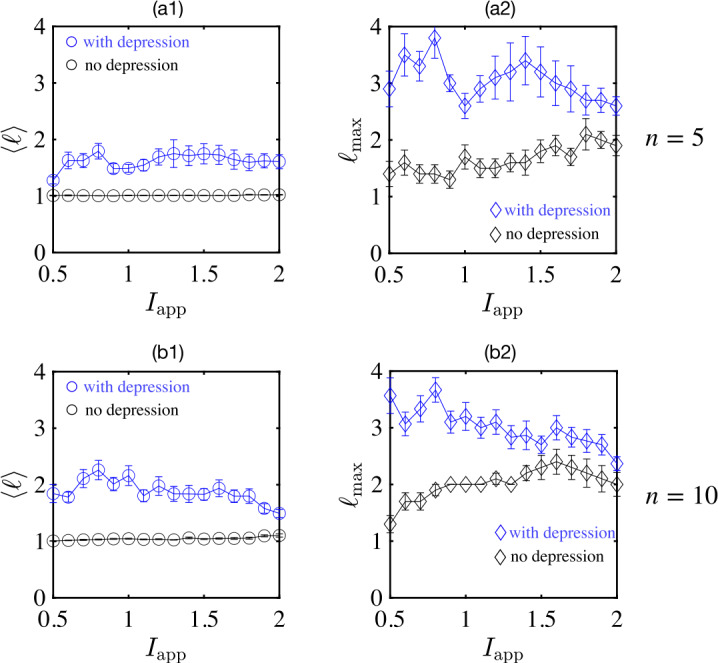
The average length $\langle \ell \rangle $ and the maximum length $\langle \ell _{\max } \rangle $ of state-transition sequences of ten randomly connected units ($\langle w_{ij} \rangle =-0.2$, ${\text{std}}(w_{ij})=1$) under repetitive stimuli with fixed duration $\tau _{{\mathrm{dur}}}=20$ and varying amplitudes $I_{{\text{app}}}$. Panels (**a1**) and (**a2**): The average length (open circle) and the max length (open diamond) of sequence in networks with (blue symbols) and without (black symbols) depression. Five ($n=5$) randomly chosen units receive the stimuli. Panels (**b1**) and (**b2**): Same as (**a1**) and (**a2**) except for all ten ($n=10$) units receiving inputs

To assess the generality of such behavior, we count the average length $\langle \ell \rangle $ as well as the maximum length $\langle \ell _{\max } \rangle $ of state-transition sequences as a function of the amplitude $I_{{\mathrm{app}}}$ of repeated stimuli of fixed duration $\tau _{{\mathrm{dur}}}=20$. The results are summarized in Fig. 7, where we compare two stimulus protocols: either five randomly chosen units receiving inputs (a1, a2) or all ten units receiving inputs (b1, b2). As before, we compare circuits with synaptic depression (blue circles) and without synaptic depression (black circles). In all cases, the network with synaptic depression achieves longer sequences of distinct states.

### Trends with increasing network size

**Figure 8:**
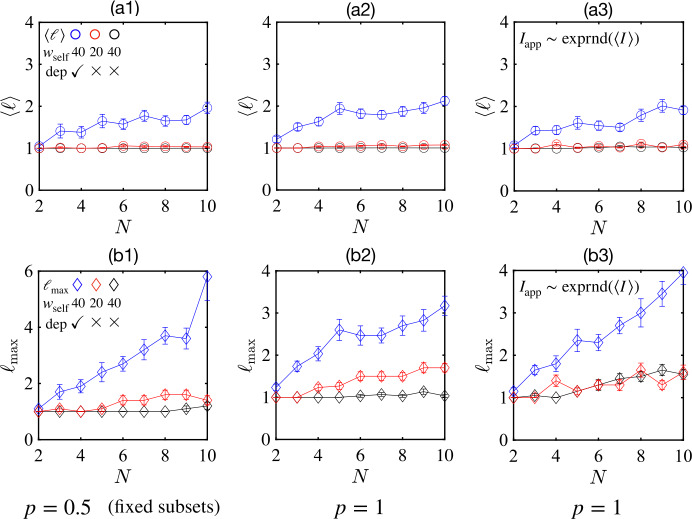
The average length $\langle \ell \rangle $ and the maximum length $\langle \ell _{\max } \rangle $ of state-transition sequences of a random network ($\langle w_{ij} \rangle =0$, ${\text{std}} (w_{ij} )=N^{-1/2}$) scale with the network size *N*. Rows 1 and 2: The average length $\langle \ell \rangle $ (open circles) and the maximum length $\langle \ell _{\max } \rangle $ (open diamonds) vs *N*. Panels (**a1**) and (**b1**): Fixed random half of units in the network ($p=0.5$) receive identical inputs. Color codes indicate combinations of self-coupling strength $w_{{\text{self}}}$ and synaptic depression. Panels (**a2**) and (**b2**): Same as (**a1**) and (**b1**) except all units in the network receive identical input ($p=1$). Panels (**a3**) and (**b3**): Same as (**a2**) and (**b2**) except that $I_{{\text{app}}}$ is drawn from an exponential distribution with the same mean as (**a2**) and (**b2**). The duration and the amplitude of stimuli are fixed ($\tau _{{\text{dur}}}=25$, $I_{{\text{app}}}=1.5$) in all cases

We have seen that synaptic depression leads to more state transitions and longer sequences for small random networks. It is tempting to explore the scaling behavior as the network size *N* increases. In Fig. 8, we estimate the average length $\langle \ell \rangle $ and the maximum length $\langle \ell _{\max } \rangle $ of the sequences of distinct states produced by repeated identical stimuli in networks of different sizes. In (a1, b1), a fixed random half of all units receive identical inputs. In (a2, b2), all units receive identical inputs. In (a3, b3), all units receive random inputs that are drawn from an exponential distribution with the same mean (equal to the constant value in a2, b2). The results are qualitatively similar in all three of these conditions of fixed input per stimulus. In all cases the inclusion of synaptic depression (blue circles) leads to longer sequences of distinct states.

Since the number of attractor states scales exponentially with *N* in the limit of low cross-coupling, one might expect the length of sequences following repeated stimuli to consistently increase with further increases in *N*. Therefore, we simulated networks with $N = 20$, $N=50$, and $N=100$ and assessed their properties by sampling initial conditions (it is not feasible to test the presence of and characterize all states when they number on the order of 2^50^ or 2^100^). While our simulations did suggest an exponentially increasing number of attractor states with increasing *N*, the transient chaos present in the large-*N* limit [38] reduces the practical use of these states for encoding sequence information. Specifically, the duration of transient dynamics increases with *N* such that, for example, with $N=100$ (and $\langle w_{ij} \rangle =0$, ${\mathrm{std}}(w_{ij})=0.1$) the majority of initial conditions did not lead to a steady state within five seconds. Therefore, while the number of distinct vectors of network firing rate could increase with successive stimuli (up to 100 or more) the firing rates were not stable, so final states depended sensitively on the interval between stimuli. Such behavior was present in networks both with and without synaptic depression, the main distinction being that larger cross-connections and larger stimuli were needed to produce dynamical responses if depression were absent.

When we only counted state sequences for which activity reached a fixed point within five seconds of each stimulus offset, we found that with an optimal strength of cross-coupling, the lengths of state sequences increased as *N* increased from 10 to 20 to 50, but then leveled out by $N = 100$. Specifically, the mean length $\langle \ell \rangle $ increased from 3.5 to 6.3, then decreased to 5.2 in the networks with depressing synapses, while the mean length increased from 2.3 to 3.4 to 4.5 in the networks without depressing synapses, as *N* increased from 20 to 50 to 100. Similarly, the across-network average of the maximum length of state sequence $\langle \ell _{\max } \rangle $ increased from 7.7 to 12.5 to 13.6 in the networks with depressing synapses and from 3.5 to 6.4 to 11.7 in the networks with depressing synapses as *N* increased from 20 to 50 to 100. However, if we removed the restriction that steady state should be reached between stimuli, or increased the delay between stimuli, sequences of distinct states of many tens in length were common (following repeated identical stimuli) by $N=100$.

## Discussion

In this paper, we consider small circuits of bistable neural populations with synaptic depression, focusing on the circuit responses to uniform stimuli with different amplitudes and durations. Because of the negative feedback generated by synaptic depression, which operates on a slow time scale in comparison to that for changes in firing rate or synaptic current, the system has an underlying oscillatory component. The oscillatory component can cause an intricate deformation of the basins of attraction that separate the fixed points where individual units are either active or inactive. The final state of the system reached after a perturbing stimulus thus sensitively depends on the properties of the stimulus.

In the absence of cross-coupling, the number of stable fixed points of the system is $2^{N}$, where *N* is the number of bistable units. While the number of stable fixed points is maximized in this limit, the lack of interaction between units means the responses to stimuli are rather limited and the history dependence is trivial. Conversely, with very strong cross-couplings, subsets of units become very highly correlated in their activity, reducing the effective *N*: for example, two units with strong reciprocal cross-excitation are always ON together or OFF together, so act together more like a single unit. We find that with weak cross-couplings, the total number of stable fixed points can remain high, while the interactions between units enables a simple, uniform stimulus (identical to all units) to cause a network response that traces a high-dimensional trajectory through the space of units’ activities. The high-dimensionality of the response leads to history dependence and richness in the types of stable states achievable by a stimulus that excites all units equally. This behavior allows networks of many units to retain separate information about the amplitude, duration, and the number of identical, repeated stimuli [24, 36].

Our work follows that of others demonstrating the richness of states in networks with coupled units. Prior work showed that in the macroscopic limit, with weak self-coupling and strong, balanced cross-coupling, a chaotic regime exists [39], whereas when the self-coupling is strong enough that each unit is bistable, multiple stable states exist and can be reached by transient chaos [38]. Here, we focused on smaller circuits and included the impact of synaptic depression, a common feature of cortical synapses. Synaptic depression can reduce the total number of fixed points by reducing the stability of the ON state (active synapses are effectively weakened by depression). However, the same effect can enhance the number of states reachable by a uniform stimulus, as a weakening of the connections within previously active units allows new units to become ON when the duration of the stimulus is extended. Similarly, such relative destabilization of previously active states enhances the history dependence of stimulus responses and causes the network’s activity to explore a wider range of the state space. We expect that incorporation of firing-rate adaptation in the neural responses would have a similar effect in destabilizing active states.

Our results show that the network responses are richer when the successive stimuli target only a subset of the units, instead of all of them. In this study we considered stimuli that target a randomly selected half of the units, with successive stimuli stimulating the same set of units in an identical manner. One may imagine that such selective targeting could reduce the overall repertoire of responses, constraining the ability of individual units to transition from an OFF state to an ON state to those units receiving the stimulus. However, the results of our single-unit studies (Figs. 1 and 2) demonstrate that excitatory input to a unit can switch it from ON to OFF as well as from OFF to ON, and the reciprocal connections within the network allow non-excited units to change their states.

The dependence of network activity on the duration of stimuli or interval between stimuli is particularly noticeable when intervals on the order of a few hundred milliseconds are present in auditory tasks. Synaptic depression operates on a suitable time scale to produce the ongoing network dynamics that could account for such interval or duration dependence [40].

While our work here focuses on the dynamics of network behavior in the presence of a stimulus which is constant in time, the dependence on initial conditions of the network’s response to a given stimulus imbues the network with history dependence. Therefore, the network can respond differently, according to the number and/or types of and/or order of preceding stimuli [24, 36, 40]. In this manner, such networks could account for the observed transitions of neural activity through a set of distinct attractor states during a counting task [36, 41] and could even provide a basis for context-dependent integration of stimulus properties [42].

## Figures and Tables

**Figure 9:**
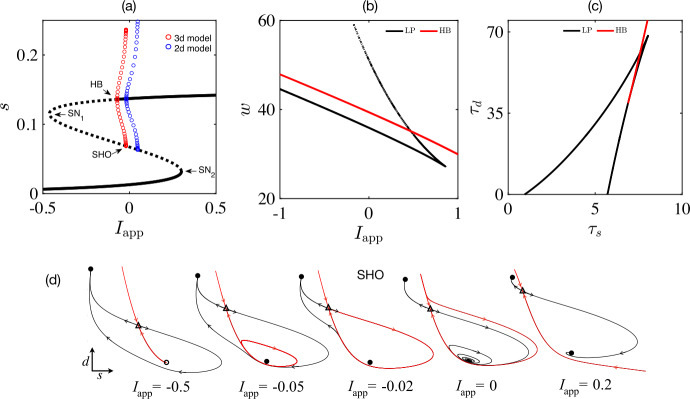
Bifurcation diagrams for a single population. (**a**) Bifurcations of the synaptic current (*s*) as a function of the stimulus ($I_{{\text{app}}}$). Two saddle-node (SN) bifurcations take place at $I_{\text{SN}_{1}}=-0.4627$ and $I_{\text{SN}_{2}}=0.3002$. An unstable limit cycle arises from a subcritical Hopf bifurcation (HB) at $I_{{\text{HB}}}= \{ -0.07069,-0.01817 \} $ for the 3d (red) and 2d (blue) model (see text). The limit cycle merges with the saddle in a saddle-homoclinic orbit (SHO) at $I_{{\text{SHO}}}= \{ -0.02013,0.04802 \} $ for the 3d and 2d models. The system is bistable between HB and SN_2_. (**b**) Two-parameter bifurcations of $I_{{\text{app}}}$ versus self-coupling weight *w*. The bistable region is between the top limit point (LP) line and the HB line. (**c**) Two-parameter bifurcations of time constants $\tau _{s}$ versus $\tau _{d}$ (both measured in $\tau _{r}$). The bistable region is between the left LP line and the HB line. A cusp point is at $(\tau _{s},\tau _{d})\approx (8.0,68.1 )$. (**d**) Bifurcation sequence in the *s*-*d* plane between SN_1_ and SN_2_. The OFF state remains stable (filled circle with small *s* and large *d*). Red (black) lines are stable (unstable) invariant manifolds of the saddle (triangle). The ON state is initially unstable (open circle) and becomes stable at the Hopf bifurcation. The central plot depicts how the unstable limit cycle terminates at the saddle when $I_{\text{app}}=I_{\text{SHO}}$

## Appendix

### Bifurcation analysis for a single unit

At a fixed point $(r,s,d )$ of a single unit, $f'=f (1-f )=r (1-r )$. The stability is captured by the Jacobian (Eq. (9)), whose eigenvalues *λ* are roots of a cubic characteristic polynomial $P (\lambda )=\lambda ^{3}+A_{2}\lambda ^{2}+A_{1} \lambda +A_{0}$ with coefficients

11 $$\begin{aligned}& A_{0} = \alpha \beta \biggl[1+ (a+b )r- \frac{bwr (1-r )}{1+ (a+b )r} \biggr], \end{aligned}$$

12 $$\begin{aligned}& A_{1} = \beta (1+ar )+\alpha \biggl[ \frac{1+ (a+b )r}{1+ar}- \frac{bwr (1-r )}{1+ (a+b )r} \biggr] \\& \hphantom{A_{1} ={}}{} +\alpha \beta \bigl(1+ (a+b )r \bigr), \end{aligned}$$

13 $$\begin{aligned}& A_{2} = 1+\beta (1+ar )+\alpha \frac{1+ (a+b )r}{1+ar}. \end{aligned}$$

Using the Routh–Hurwitz criterion [43], the fixed point is stable ($\operatorname{Re}\lambda <0$) if $A_{0}, A_{2}>0$ and $A_{1}A_{2}-A_{0}\equiv H_{2}>0$. When an eigenvalue becomes zero ($A_{0}=0$) or purely imaginary ($H_{2}=0$ and $A_{0}, A_{2}>0$), the fixed point undergoes a saddle-node (SN) or a Hopf bifurcation (HB).

The condition for a saddle-node bifurcation ($A_{0}=0$) is equivalent to

14 $$ w= \frac{ (1+ (a+b )r )^{2}}{br (1-r )}. $$

Since $\dot{r}=0$, $r=f (ws-\theta +I )$. This implies that *θ* must satisfy

15 $$ \theta =\frac{1+ (a+b )r}{1-r}-\ln \frac{r}{1-r}+I. $$

Graphing *w* and *θ* as two parametric equations in *r* gives the boundary of a bistable region in the *w*-*θ* plane [black solid lines in Fig. 5(a)]. Similar wedge boundaries were found in [44] for rate models without synaptic depression.

The boundary lines terminate at a cusp

16 $$ (w_{c},\theta _{c} )= \bigl(4 (a+b+1 )/b,2+ \ln (a+b+1 )+I \bigr), $$

where a co-dimension-2 bifurcation takes place. The wedge has a width

17 $$ \Delta \theta (w )=4w_{c}^{-1}\sqrt{w (w-w_{c} )}+\ln \frac{2w-w_{c}-2\sqrt{w (w-w_{c} )}}{2w-w_{c}+2\sqrt{w (w-w_{c} )}} $$

which scales linearly with *w* when $w\gg w_{c}$. Thus it is easier to obtain bistability with stronger self-excitation.

Since $A_{2}$ is always positive, the condition for a Hopf bifurcation ($A_{1}A_{2}=A_{0}>0$) leads to $w=P (r )/Q (r )\equiv w_{{\mathrm{HB}}}$$(r )$ with

18 $$\begin{aligned}& P (r ) = \bigl(1+ (a+b )r \bigr) \bigl(1+ \beta (1+ar ) \bigr) \bigl[1+ \alpha +r \bigl(a+\alpha (a+b ) \bigr) \bigr] \\& \hphantom{P (r ) ={}}{}\times\bigl(\alpha +\alpha (a+b )r+\beta (1+ar )^{2} \bigr), \end{aligned}$$

19 $$\begin{aligned}& Q (r ) = \alpha br (1-r ) (1+ar ) \bigl[1+\alpha +r \bigl(a^{2} \beta r+a (1+\beta )+ \alpha (a+b ) \bigr) \bigr]. \end{aligned}$$

Fixing *θ* and treating $I_{{\mathrm{app}}}$ as a parameter, we get an equation for $I_{{\mathrm{app}}}$ at the Hopf bifurcation:

20 $$ I_{{\mathrm{app}}} (r )=\ln \frac{r}{1-r}-w_{{\mathrm{HB}}} (r )s (r )+ \theta . $$

Figure 9(a) illustrates bifurcations of the synaptic current *s* as a function of the applied stimulus $I_{{\mathrm{app}}}$ (with *w* and *θ* fixed): For large inhibitory input, the OFF state is the only attractor. An unstable ON state and a saddle point emerge from a saddle-node (SN) bifurcation when $I_{{\mathrm{SN}}_{1}}\approx -0.46$. The ON state tunes stable when a subcritical Hopf bifurcation (HB) takes place at $I_{{\mathrm{HB}}}\approx -0.07$, which gives rise to an unstable limit cycle around the ON state. When $I_{{\mathrm{SHO}}}\approx -0.02$, this limit cycle merges with the saddle point via a saddle-homoclinic orbit (SHO). The system is bistable at zero input and remains so until another SN bifurcation at a larger excitation ($I_{{\mathrm{SN}}_{2}}=0.3$). Note that oscillatory solutions stem from the slow feedback of the depression.

We separate the fast and the slow variables by assuming *r* reaches a steady value given a synaptic current *s*, $r\approx \bar{r} (s )=f (ws-\theta +I )$. The reduced model (*s* vs *d*) captures the full model’s dynamics except for slightly shifted Hopf and SHO bifurcations ($I_{{\mathrm{HB}}}\approx -0.02$ and $I_{{\mathrm{SHO}}}\approx 0.05$). Thus to determine the system’s state under constant input, it is sufficient to study the *s*-*d* model.

In Fig. 9(b), we plot $w_{HB}$ and $I_{{\mathrm{app}}}$ as parametric functions in *r*. The bistable region is above the HB curve (red line) and below the upper boundary of the LP curve (black line). The wedge region ends at a cusp point $(w_{c},I_{c} )= (4b^{-1} (a+b+1 ), \theta -\ln (a+b+1 )-2 )$.

Figure 9(c) shows the fixed point’s bifurcation with varying time constants $\tau _{s}$ and $\tau _{d}$, which has similar wedge-shaped structure as in Fig. 9(a). The region between the left boundary of the limit point (LP) curve and the HB curve supports bistable solutions. Hence the bistability is robust for slow depression and a wide range of synaptic time constants.

Figure 9(d) graphs the stable states and manifolds of fixed points in the *s*-*d* plane. As the input increases, the basin of attraction of the ON state grows quickly. A strong excitatory stimulus may kick the system near the ON state. When the input shuts off, the vector fields and basins of attraction all resume to the case with $I_{\mathrm{app}}=0$. Then the system’s instantaneous location in the *s*-*d* plane may be in the small basin of the ON state or the large basin of the OFF state, leading to distinct final states. Similar rebound behavior exists in conductance-based models with slow calcium channels [43] and is a generic feature in fast-slow systems [45]. The deformed basins of attraction due to slow depression result in history-dependent responses under stimulations.

## Footnotes

1 We choose a rate vector $r_{0}= (r_{1},r_{2} )^{T}$ and set the initial condition as $(r_{0},s (r_{0} ),d (r_{0} ) )$, then label the final state after integrating the differential equations for a long time.

2 We use a binary code to represent the state vector: 0 and 1 stand for the OFF state and ON state respectively, with firing rates $r_{{\mathrm{OFF}}}\approx 0.01$ and $r_{{\mathrm{ON}}}\approx 0.6$.

## Abbreviations

HB: Hopf bifurcation
LP: Limit point
SN: Saddle-node
SHO: Saddle-homoclinic orbit
